# Does Mirikizumab benefit patients with plaque psoriasis? a systematic review and meta-analysis

**DOI:** 10.1007/s00210-025-04416-0

**Published:** 2025-07-10

**Authors:** Shrouk F. Mohamed, Mohamed R. Abdelraouf, Mohamed Wagdy, Ahmed Hegazy, Mena Essam, Ahmed Elsekaily

**Affiliations:** 1 Alexandria Faculty of Medicine, Alexandria, Egypt; 2 Faculty of Medicine, Modern University for Information and Technology, Cairo, Egypt

**Keywords:** Plaque psoriasis, Mirikizumab, Monoclonal antibody

## Abstract

**Background:**

Plaque psoriasis is a chronic skin condition characterized by skin scales. It causes a cosmetic problem for patients. Mirikizumab has shown promising results in managing psoriasis. Nevertheless, there remains a dispute over its safety. This systematic review and meta-analysis aimed to assess the effectiveness of mirikizumab in individuals with plaque psoriasis.

**Methods:**

We searched four databases: PubMed, Scopus, Cochrane CENTRAL, and Web of Science. We included trials comparing mirikizumab with placebo in psoriatic patients. Two independent authors extracted data in an Excel sheet. We used RevMan software (version 5.2) to perform meta-analysis.

**Results:**

A meta-analysis of three RCTs with a total of 1648 patients was performed. Mirikizumab showed significantly higher rates of PASI 75, PASI 90, and PASI 100 responses at week 16 compared to placebo, with risk ratios (RR) of 10.47, 11.5, and 25.94, respectively (all P < 0.00001). According to adverse events, we found no difference between mirikizumab and placebo. Furthermore, mirikizumab showed significant results compared to placebo in terms of DLQI, sPGA, and body surface area, with P < 0.00001. Unfortunately, there was no difference between the two groups regarding adverse events, with p = 0.63.

**Conclusion:**

Mirikizumab showed good responses in managing plaque psoriasis. However, additional clinical trials are required to verify its safety and evaluate its long-term efficacy.

**Supplementary Information:**

The online version contains supplementary material available at 10.1007/s00210-025-04416-0.

## Introduction

Plaque psoriasis is a chronic, immune-mediated skin disease that affects approximately 2–3% of the global population. Among its several clinical variants, plaque psoriasis (psoriasis vulgaris) is the most common form, accounting for about 80–90% of all psoriasis cases. It is characterized by well-demarcated, erythematous plaques with silvery-white scales, typically appearing on the scalp, elbows, knees, and lower back (Raharja et al. 2021; Gisondi et al. 2020). The pathophysiology is still unknown, but it is thought to be a T cell-mediated immunological reaction. Lymphocytes induce specific cytokines such as IL-12, IL-17, and IL-23, which trigger immunological responses to produce more keratinocytes (Raharja et al. 2021; Cherubin et al. 2023; Rendon and Schäkel 2019).

Furthermore, it is thought that many different genetic and environmental factors may contribute to this condition. For example, obesity, smoking, and alcohol drinking are some of the risk factors for psoriasis (Raharja et al. 2021; Salimi et al. 2020). It not only impairs quality of life due to discomfort and cosmetic concerns but is also frequently linked with systemic comorbidities, including psoriatic arthritis, cardiovascular disease, metabolic syndrome, and other related conditions, complicating its overall management.

Moreover, the chronic nature of the disease and the high costs associated with long-term treatment, particularly with biologics, place a considerable financial burden on patients and healthcare systems (Raharja et al. 2021).

Management of psoriasis requires a tailored, multidisciplinary approach. While mild cases may respond to topical agents such as corticosteroids, vitamin D analogs, and retinoids, moderate to severe cases often necessitate systemic therapies. These include conventional agents like methotrexate and cyclosporine, small-molecule inhibitors such as apremilast, and biologic therapies that target specific immune pathways, most notably the IL-17 and IL-23 axes (Sbidian et al. 2020; Chat et al. 2020).

Among the newer biologics, mirikizumab (Miri) is a humanized IgG4 monoclonal antibody used to target immunological reactions. It is composed of two subunits: p19 and p40, with the p19 subunit being unique to IL-23 and critical for its biological activity. IL-23 promotes the expansion and survival of Th17 cells, a subset of T-helper cells that produce pro-inflammatory cytokines such as IL-17A, IL-17F, and IL-22. These cytokines play a central role in driving keratinocyte hyperproliferation, sustained skin inflammation, and the characteristic psoriatic plaques. Overexpression of IL-23, particularly the p19 subunit, has been detected in psoriatic skin lesions, and its signaling is strongly associated with disease severity. Miri has shown incredible results in immune-mediated inflammatory diseases, such as ulcerative colitis, which makes it vulnerable to being tried in other conditions. Also, it was associated with fewer adverse effects and better efficacy. A few clinical trials evaluating patients with varying degrees of plaque psoriasis found rapid and long-lasting clinical improvement, along with a decrease in Psoriasis Area and Severity Index (PASI) scores (Blauvelt et al. 2022 Dec 1; Reich et al. 2019 Jul 1; Papp et al. 2023; Magro et al. 2024; Kobayashi et al. 2024).

This study aimed to assess the effectiveness of mirikizumab in individuals with plaque psoriasis.

## Methods

This study followed the PRISMA (Preferred Reporting Items for Systematic Reviews and Meta-Analyses) guidelines for systematic review and meta-analysis, ensuring transparency and thoroughness in reporting the research. The protocol was registered on PROSPERO (Moher et al. 2009), available from https://www.crd.york.ac.uk/PROSPERO/view/CRD42024578351

### Search strategy

We investigated several databases, including PubMed, Cochrane CENTRAL, Scopus, and Web of Science (WOS), for relevant studies using the following search strategy: “((LY3074828 OR miri OR mirikizumab) AND (psoriasis OR Palmoplantaris Pustulosis OR Pustulosis of Palms and Soles))”.

### Eligibility criteria

- Language: English studies.
- Population: Patients who presented with demarcated erythematous plaques with silvery-white scales, commonly located on the scalp, elbows, knees, and lower back, and a diagnosis of plaque-psoriasis was confirmed by a dermatologist.
- Intervention: Use of Mirikizumab.
- Comparator: Placebo.
- Outcome Measures: PASI, body surface area (BSA), Dermatology Life Quality Index (DLQI), static Physician’s Global Assessment (PGA), and any reported adverse effects.
- Study design: All randomized clinical trials and observational research, whether case–control or cohort studies.

We excluded reviews, case studies, editorial letters, conference abstracts, study protocols, animal studies, and studies with insufficient data from our analysis.

### Study selection

After abstract and title screening, we performed full-text screening. Two independent authors carried out the screening process by applying exclusion and inclusion criteria. Two authors extracted data from three included trials, which consisted of information on the study ID, type of study, number of patients recruited, duration, criteria for inclusion, average ages, age at diagnosis, results, and interventions used. Any conflicts were resolved through a group discussion.

### Outcome assessment

The primary outcomes were PASI response, DLQI, BSA, and sPGA. The secondary outcomes included the overall count of adverse events (AEs), infections, malignancies, upper respiratory tract infections, medication discontinuation, and injection site reactions.

### Quality assessment

We evaluated the quality of randomized controlled trials (RCTs) utilizing the Cochrane Handbook (ROB-2), taking into account the following criteria for study quality: randomization, allocation concealment, blinding, missing data, selective outcome reporting, and other biases.

### Statistical analysis

We did our meta-analysis using the Review Manager (RevMan) software version 5.2. We used risk ratios for dichotomous data and mean differences for continuous data with 95% confidence intervals. We used the chi-square test to calculate I^2^ and assess heterogeneity. Subgrouping analysis was done based on drug dosage.

## Results

### Literature search

**Fig. 1:**
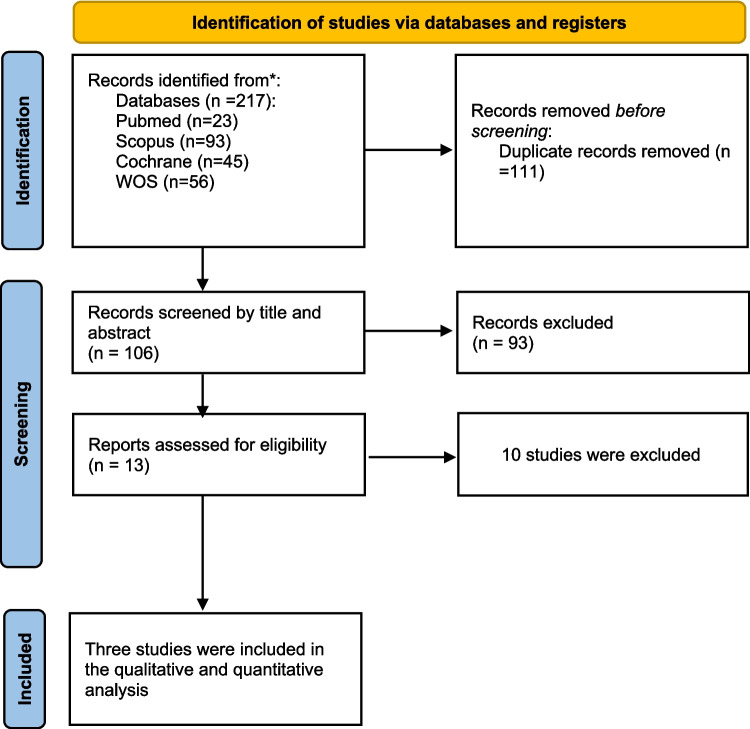
Prisma Flow Diagram

A total of 217 articles were identified through searching different databases. By looking at the titles and abstracts of 166 records, 111 records were duplicates. The full-text screening was done for 13 eligible studies. Finally, three papers met our inclusion criteria (Blauvelt et al. 2022 Dec 1; Reich et al. 2019 Jul 1; Papp et al. 2023). The PRISMA flowchart is shown in Fig. 1.

### Characteristics of the included studies

Three RCTs involved participants with plaque psoriasis over the age of 40. Table 1 shows the general characteristics of the included studies, while Table 2 outlines the extracted data of the participants'baseline characteristics.

**Table 1:** Summary of included studies

Study ID	Country	Inclusion and Exclusion	Key Findings
Blauvelt 2022	Germany, Japan, South Korea, Mexico, Poland, Russian Federation, Taiwan, USA, Puerto Rico	**Exclusion:** Uncontrolled or unstable health conditions at screening (e.g., cerebrocardiovascular, respiratory, hepatic, renal, gastrointestinal, endocrine, hematological, neurological disease); abnormal laboratory values; recent treatment with systemic anti-infective medications; recent use of anti-TNF, anti-IL-17 biologics; or previous use of biologics targeting IL-12/23 or IL-23	Mirikizumab was superior to placebo at week 16 and maintained efficacy through week 52 with no new safety signals
Reich 2019	Canada, Germany, Japan, Poland, USA	**Inclusion:** Adults aged 18–75 years with chronic plaque psoriasis for ≥ 6 months, ≥ 10% BSA involvement, PASI score ≥ 12, sPGA score ≥ 3, eligible for biological therapy **Exclusion:** Recent use of anti-TNF, anti-IL-17 biologics, or previous exposure to biologics targeting IL-23 (except briakinumab)	At week 16, 67% of patients treated with mirikizumab 300 mg at 8-week intervals achieved PASI 90. Similar rates of treatment-emergent adverse events were reported in both the mirikizumab and placebo groups
Papp 2023	Argentina, Australia, Canada, Czech Republic, France, Germany, Hungary, Israel, Italy, Japan, South Korea, Poland, Puerto Rico, Spain, UK, USA	**Inclusion:** Adults aged ≥ 18 years with chronic plaque psoriasis for ≥ 6 months, ≥ 10% BSA involvement, PASI score ≥ 12, sPGA score ≥ 3, candidates for systemic therapy or phototherapy **Exclusion:** Uncontrolled or unstable health conditions at screening (e.g., cerebro-cardiovascular, respiratory, hepatic, renal, gastrointestinal, endocrine, hematological, neurological disease); abnormal laboratory values	This trial showed superiority of mirikizumab 250 mg over placebo in patients with moderate-to-severe plaque psoriasis, with a safety profile consistent with that of the IL-23 class

**Table 2:** Baseline characteristics of included studies

Study ID	Groups	Age Mean (SD)	Sex Male n(%)	BMI Mean (SD)	PASI Mean (SD)	PSS Mean (SD)	DLQI Mean (SD)	Duration of Psoriasis Mean (SD)	N. of Patients Took Previous Biological Therapy n(%)	N. of Patients Took Previous Systemic Therapy n(%)	Patients with Psoriatic Arthritis n(%)
**Papp, 2023**	250 mg Mirikizumab	45.8 (13.0)	296 (65.2%)	29.8 (7.5)	21.1 (8.3)			17.2 (12.5)	72 (15.9%)		42 (9.3%)
	Placebo	43.8 (12.5)	82 (73.2%)	29.5 (5.3)	21.8 (8.6)			18.8 (12.7)	102 (91.1%)	48 (42.9%)	7 (6.3%)
**Reich, 2019**	Mirikizumab	47.6 (13.2)	110 (71.9%)	29.8 (5.9)	19.9 (7.8)	48.7 (17.75)	12.6 (6.5)	19.03 (12.49)	63 (41.2%)	120 (78.4%)	
	Placebo	46 (12.4)	42 (81%)	29.2 (5.8)	19.7 (7.4)	50.4 (19.3)	14.1 (7.2)	18 (9.8)	21 (40%)	38 (73%)	
**Blauvelt, 2022**	Mirikizumab 250 mg	46.4 (13.6)	299 (70.7%)		22.3 (9.8)	23.3 (10.5)	14.4 (7.4)	17.7 (11.5)	139 (32.9%)		69 (16.3%)
	Placebo	45.7 (13.7)	74 (69.2%)		23.5 (10.1)	22.4 (10.7)	13 (7)	17.0 (10.9)	31 (29%)		8 (7.5%)

### Quality assessment

**Fig. 2:**
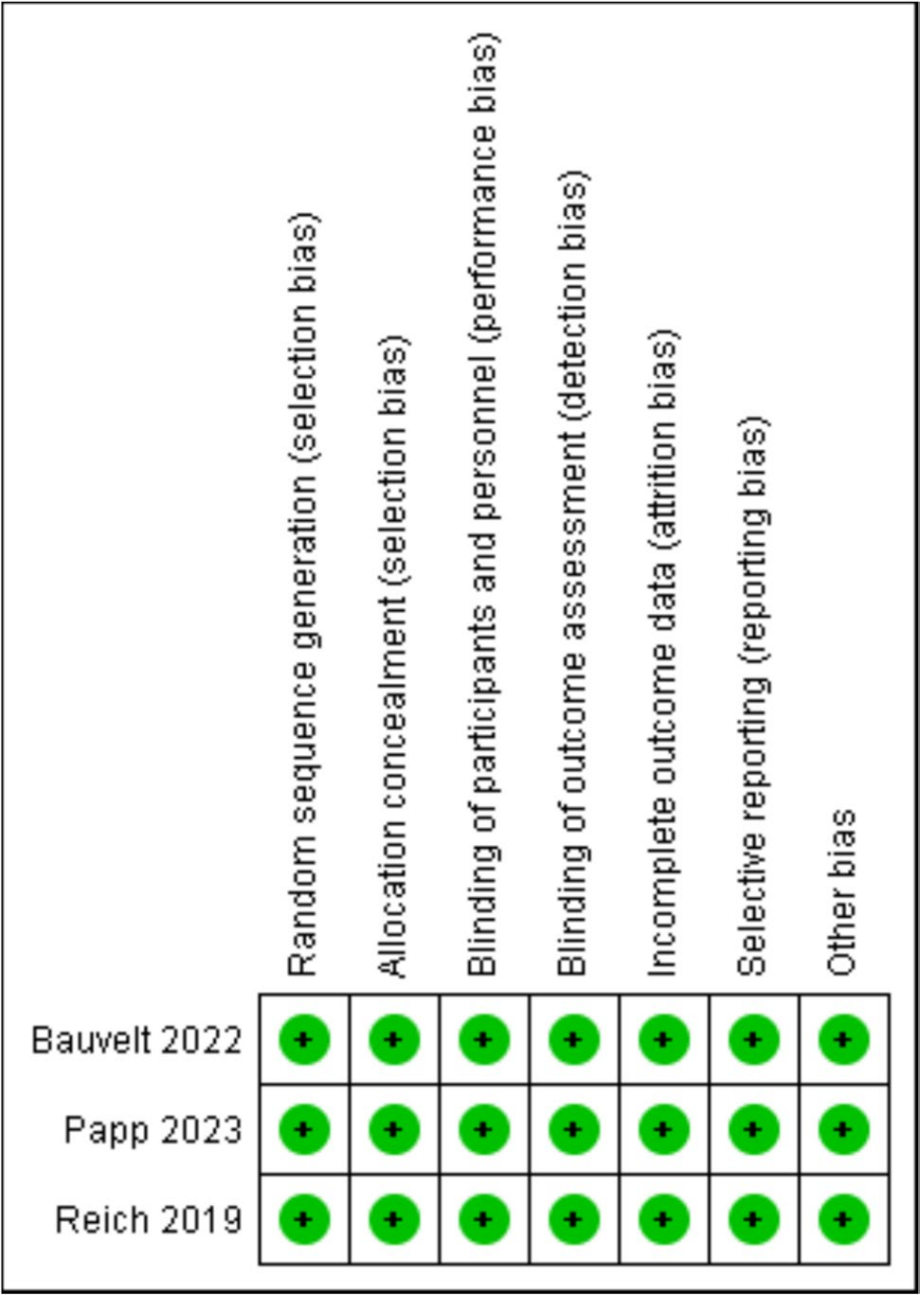
ROB summary

**Fig. 3:**
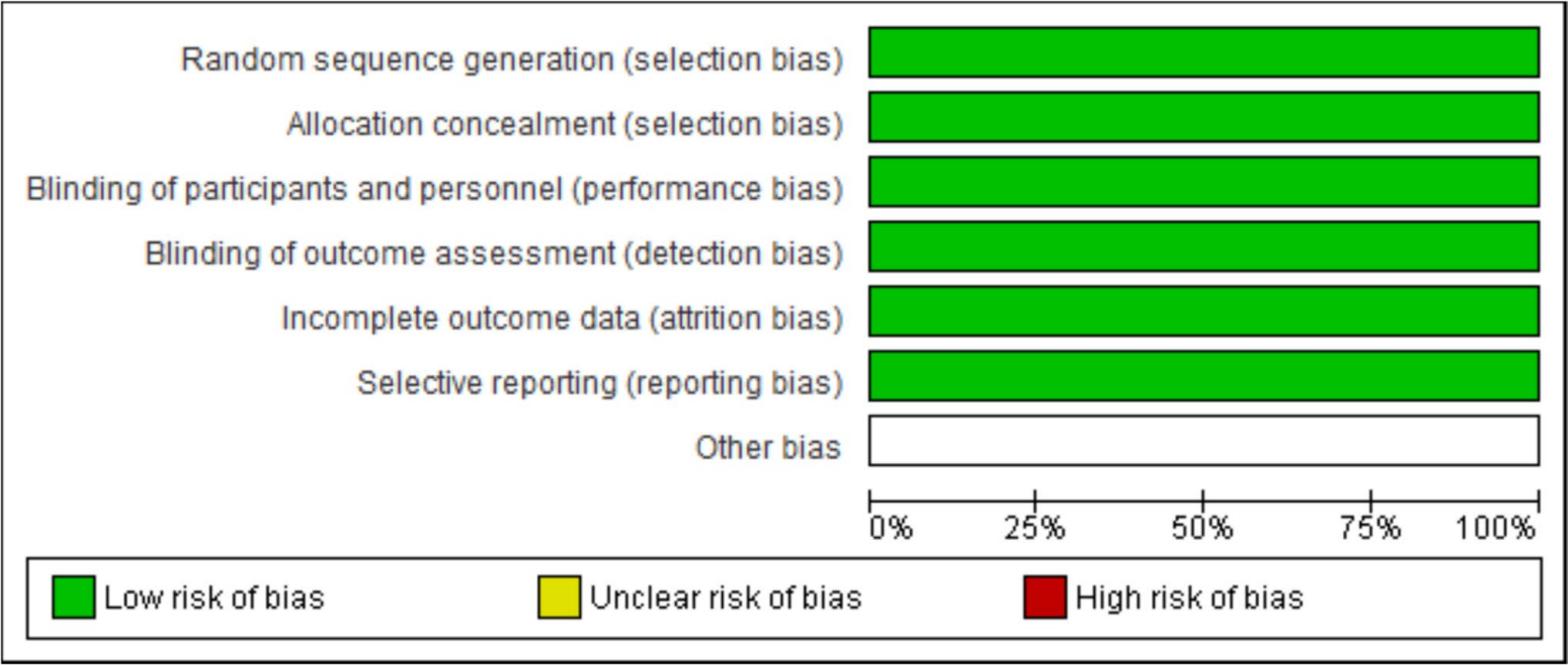
ROB Graph

ROB-2 evaluation of RCTs found that the three included studies demonstrated high quality, as shown in Fig. 2 and Fig. 3.

### Outcomes

#### Psoriasis area and severity index (PASI 75, responders) at week 16

**Fig. 4:**
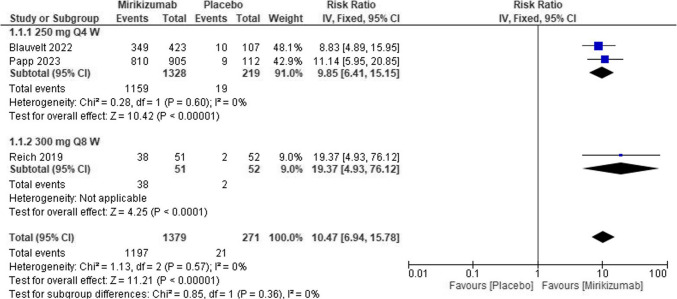
Forest plot of PASI 75 in the mirikizumab group vs. placebo

Mirikizumab treatment significantly increased the risk of obtaining PASI by 75% compared to placebo (RR = 10.47, 95% CI (6.94, 15.78), P < 0.00001). The pooled analysis was homogeneous (I^2^ = 0%). Figure 4

#### Psoriasis area and severity index (PASI 90, responders) at week 16

**Fig. 5:**
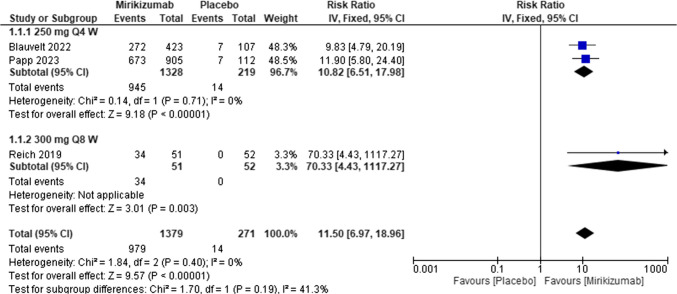
Forest plot of PASI 90 in the mirikizumab group vs. placebo

The overall effect showed that patients treated with Miri had a markedly higher likelihood of achieving PASI 90% compared to placebo [RR = 11.5, 95% CI (6.97, 18.96), P < 0.00001]. The analysis was homogeneous (I^2^ = 0%; P for Cochran Q = 0.40). Figure 5

#### Psoriasis area and severity index (PASI 100, responders) at week 16

**Fig. 6:**
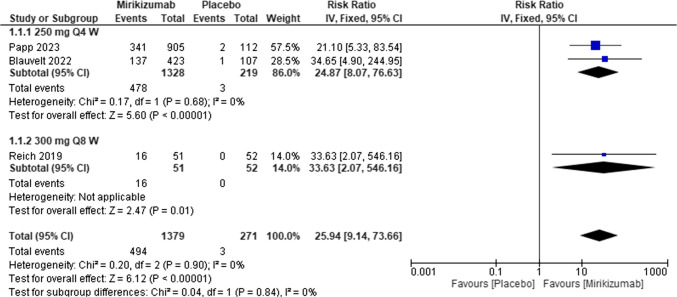
Forest plot of PASI 100 in the mirikizumab group vs. placebo

Miri produced substantially more significant improvements in PASI 100 compared to placebo [RR = 25.94, 95% CI (9.14, 73.66), P < 0.00001]. There was no heterogeneity among the included studies (I^2^ = 0%; P for Cochran Q = 0.90). Figure 6

#### Dermatology life quality index DLQI (0,1)

DLQI was reported by two studies showing that mirikizumab was associated with higher DLQI compared to placebo [RR = 10.94, 95% CI (5.12, 22.03), P < 0.00001]. There was no heterogeneity among the included studies (I^2^ = 0%; *P* = 0.81). (S1 in Supplements).

#### Static physician’s global assessment sPGA score

**Fig. 7:**
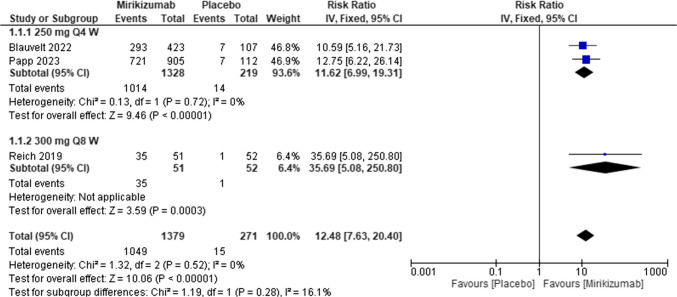
Forest plot of static Physician’s Global Assessment in the mirikizumab group vs. placebo

Patients treated with mirikizumab had a higher sPGA score than patients managed with a placebo [RR = 12.48, 95% CI (7.63, 20.40), P < 0.00001]. There was no heterogeneity among the included studies (I^2^ = 0%; *P* for Cochran Q = 0.52). Figure 7

#### Body surface area (BSA)

The results found that patients receiving mirikizumab were much more likely to attain better BSA outcomes than those on placebo [RR = 34.53, 95% CI (13.03, 91.51), P < 0.00001]. The pooled data were homogenous (I^2^ = 0%; P = 0.89). (S2 in Supplements).

#### Overall adverse events

We found no significant differences in overall adverse events between the two groups [RR = 0.97, 95% CI (0.86, 1.1), P = 0.63]. No heterogeneity was found among the included studies (I^2^ = 0%; P for Cochran Q = 0.96). (S3 in Supplements).

#### Discontinuation from treatment due to AEs

The overall results showed that mirikizumab did not considerably lead to discontinuing treatment compared to placebo [RR = 0.68, 95% CI (0.14, 3.2), P = 0.63]. The analysis was consistent (I^2^ = 0%; P for Cochran Q = 0.90). (S4 in Supplements).

#### Infection

The risk of infection did not differ between the two regimens [RR = 1.11, 95% CI (0.89, 1.38), P = 0.36]. The included studies were homogenous (I^2^ = 0%; P for Cochran Q = 0.41). (S5 in Supplements).

#### Upper respiratory tract infection

Mirikizumab did not increase the risk of URTI compared to placebo [RR = 1.61, 95% CI (0.85, 3.04), P = 0.14]. The analysis was consistent (I^2^ = 0%; P for Cochran Q = 0.60). (S6 in Supplements).

#### Malignancies

Treating psoriatic patients with Mirikizumab did not reveal an increase in the risk of malignancy compared to placebo [RR = 0.45, 95% CI (0.06, 3.40), P = 0.44]. No heterogeneity was found (I^2^ = 0%; P for Cochran Q = 0.55). (S7 in Supplements).

#### Injection site reaction

The overall effect revealed that injection site reaction was similar between the two interventions [RR = 1.22, 95% CI (0.68, 2.19), P = 0.51]. The pooled analysis was homogeneous (I^2^ = 0%; P for Cochran Q = 0.67). (S8 in Supplements).

## Discussion

Psoriasis is considered one of the emerging skin disorders that affects patients’ lives. Nowadays, biological therapies have shown significant results in managing psoriasis. Different types of biologics showed promising results in plaque psoriasis, besides arthritic psoriasis, such as guselkumab, risankizumab, and tildrakizumab, which target the same pathway (Raharja et al. 2021; Salimi et al. 2020; Liu et al. 2020; Menter et al. 2021).

Our results consistently demonstrate that Miri is highly effective in managing psoriasis at 16 weeks, with many patients achieving PASI 75, PASI 90, PASI 100, BSA, and DLQI than placebo.

Nevertheless, there is a high potential for developing malignancies and continuous infections due to its immunosuppression effect; our results revealed no difference between placebo and Miri in developing infections or malignancies.

Another systematic review and meta-analysis that evaluated the safety and efficacy of IL-17, IL-12/23, and IL-23 inhibitors in the management of moderate to severe plaque psoriasis produced similar findings (Bilal et al. 2018).

Previous studies on guselkumab and risankizumab also reported strong PASI 90 responses in moderate-to-severe psoriasis. Several studies on guselkumab demonstrated a significant proportion of patients attained PASI 90, which is comparable to the results seen with mirikizumab (Blauvelt et al. 2017; Reich et al. 2017).

The rapid response with mirikizumab also parallels earlier findings from trials on risankizumab. Both drugs, through selective IL-23 inhibition, offer rapid reductions in psoriasis symptoms, with many patients experiencing significant improvement within the first 12 weeks. In the OASIS-1 study, results showed that mirikizumab effectively cleared skin plaques within 16 weeks and maintained this improvement for up to 52 weeks in psoriasis patients. Also, it is important to achieve patient satisfaction (Blauvelt et al. 2022 Dec 1; Gordon et al. 2018).

Mirikizumab is considered one of the broader treatments for plaque psoriasis and personalized therapies. Other biologics, like IL-17 and TNF inhibitors (adalimumab, infliximab, secukinumab, ixekizumab, etc.), revolutionized psoriasis treatment by offering more specific immune modulation compared to traditional systemic therapies like methotrexate or cyclosporine. However, the recent IL-23 inhibitors, including mirikizumab, offer even more precise targeting of the inflammatory pathways directly implicated pathophysiology of psoriasis, leading to improved efficacy and fewer adverse effects (Salimi et al. 2020; Papp et al. 2023; Bilal et al. 2018; Gordon et al. 2018).

Papp et al. compared Miri with secukinumab; they found that Miri was better than secukinumab at week 16, achieving a PASI 90 response in 74.4% and 72.8%, respectively (Papp et al. 2023).

Previous studies have shown that IL-17 inhibitors also achieve rapid skin clearance, but their mechanism targets the end effector of the IL-23/IL-17 pathway. IL-23 inhibitors, like Miri, block the upstream of cytokines, potentially offering more sustained efficacy with fewer immune-related side effects, which has led to increased interest in IL-23 inhibitors for long-term psoriasis management, with some evidence suggesting a lower relapse rate compared to IL-17 inhibitors (Liu et al. 2020; Menter et al. 2021).

Our meta-analysis has numerous strengths, and it is the first meta-analysis to assess the efficacy of Miri in plaque psoriasis. First, we included studies that were of high quality. Additionally, the consistency of results indicated by pooling data homogeneity strengthens the conclusions’ validity, making them more generalizable across populations. Besides, the study specifically addressed different outcomes, thus providing a detailed review regarding the drug.

However, despite these advantages, several limitations must be recognized. For example, the first limitation could be seen in having only three RCTs for analysis. Even though pooled analyses showed homogeneity, concerns were raised because only a few papers were considered in this analysis, which may raise some publication bias.

There were numerous abstracts discussing the efficacy of mirikizumab, but full texts have not been published yet.

In addition, our analysis focused mainly on short-term outcomes at 16 weeks, meaning we never took care of the long-term safety and efficacy of miri due to incomplete data and different dosages.

Subgroup analyses based on the dosage further supported its efficacy, with notable improvements observed in PASI scores favoring Miri over the placebo.

Regarding safety outcomes, it demonstrated no significant differences in adverse events, highlighting its favorable safety profile in comparison to the placebo.

Based on our findings, several recommendations can be made for future research and clinical practice. First, we recommend conducting large-scale RCTs with standardized protocols and more extended follow-up periods, which can provide more definitive insights into mirikizumab's long-term efficacy and safety profile.

Additionally, exploring patient-specific factors, such as genetic markers or biomarkers, may help identify subpopulations that are more likely to benefit from the therapy, leading to personalized treatment approaches. Also, studies have been conducted that compare mirikizumab and IL-17/other IL-23 inhibitors.

## Conclusion

This study showed that mirikizumab is safer and more effective in patients with plaque psoriasis than placebo. Adverse events were not of significant value with mirikizumab. However, it is still unclear if the drug will be safe in the long term. Finally, mirikizumab significantly improves the PASI score, body surface area, and DLQI at 16 weeks. We recommend further studies to demonstrate the safety of mirikizumab in psoriasis patients, focusing on determining the ideal dosage, exploring potential supplement combinations, and evaluating any potential side effects for a more thorough understanding of the treatment.

## Supplementary Information

Below is the link to the electronic supplementary material.

ESM 1 (413 KB ZIP)

## Data Availability

All source data for this work (or generated in this study) are available upon reasonable request.

## References

[CR1] Bilal J, Berlinberg A, Bhattacharjee S, Trost J, Riaz I Bin, Kurtzman DJB (2018) A systematic review and meta-analysis of the efficacy and safety of the interleukin (IL)-12/23 and IL-17 inhibitors ustekinumab, secukinumab, ixekizumab, brodalumab, guselkumab and tildrakizumab for the treatment of moderate to severe plaque psoriasis. J Dermatol Treatment [Internet]. 29(6):569–78. Available from: 10.1080/09546634.2017.1422591 10.1080/09546634.2017.1422591 29532693

[CR2] Blauvelt A, Kimball AB, Augustin M, Okubo Y, Witte MM, Capriles CR et al (2022) Efficacy and safety of mirikizumab in psoriasis: results from a 52-week, double-blind, placebo-controlled, randomized withdrawal, phase III trial (OASIS-1)*. Br J Dermatol 187(6):866–877 35791755 10.1111/bjd.21743 PMC10087045

[CR3] Blauvelt A, Papp KA, Griffiths CEM, Randazzo B, Wasfi Y, Shen YK et al (2017) Efficacy and safety of guselkumab, an anti-interleukin-23 monoclonal antibody, compared with adalimumab for the continuous treatment of patients with moderate to severe psoriasis: Results from the phase III, double-blinded, placebo- and active comparator–controlled VOYAGE 1 trial. J Am Acad Dermatol [Internet]. 76(3):405–17. Available from: 10.1016/j.jaad.2016.11.041 10.1016/j.jaad.2016.11.041 28057360

[CR4] Chat VS, Kearns DG, Uppal SK, Han G, Wu JJ (2022) Management of Psoriasis With Topicals: Applying the 2020 AAD-NPF Guidelines of Care to Clinical Practice. Cutis. Frontline Medical Communications Inc. 110:8–14. 10.12788/cutis.0573 36219602

[CR5] Cherubin M, Tebar WR, Meneghini V, Bensenor IM (2023) Psoriasis and associated risk factors: a cross-sectional analysis of the Brazilian Longitudinal Study of Adult Health. Rev Assoc Med Bras 69(6):e20230038. Available from: 10.1590/1806-9282.20230038 10.1590/1806-9282.20230038 PMC10305825 37377285

[CR6] Gisondi P, Bellinato F, Girolomoni G (2020) Topographic Differential Diagnosis of Chronic Plaque Psoriasis: Challenges and Tricks. J Clin Med [Internet]. 9(11). Available from: https://www.mdpi.com/2077-0383/9/11/3594 10.3390/jcm9113594 PMC7695211 33171581

[CR7] Gordon KB, Strober B, Lebwohl M, Augustin M, Blauvelt A, Poulin Y et al (2018) Efficacy and safety of risankizumab in moderate-to-severe plaque psoriasis (UltIMMa-1 and UltIMMa-2): results from two double-blind, randomised, placebo-controlled and ustekinumab-controlled phase 3 trials. Lancet [Internet]. 392(10148):650–61. Available from: 10.1016/S0140-6736(18)31713-6 10.1016/S0140-6736(18)31713-6 30097359

[CR8] Kobayashi T, Matsuoka K, Watanabe M, Hisamatsu T, Hirai F, Milata J et al (2024) Efficacy and safety of mirikizumab as induction and maintenance therapy for Japanese patients with moderately to severely active ulcerative colitis: a subgroup analysis of the global phase 3 LUCENT-1 and LUCENT-2 studies. Intest Res 22(2):172–185 38720466 10.5217/ir.2023.00043 PMC11079516

[CR9] Liu T, Li S, Ying S, Tang S, Ding Y, Li Y et al (2020) The IL-23/IL-17 pathway in inflammatory skin diseases: from bench to bedside. Front Immunol. Available from: 10.3389/fimmu.2020.594735 10.3389/fimmu.2020.594735 PMC7705238 33281823

[CR10] Magro F, Protic M, De Hertogh G, Chan LS, Pollack P, Jairath V, et al (2024) Effects of Mirikizumab on Histologic Resolution of Crohn’s Disease in a Randomized Controlled Phase 2 Trial. Clinical Gastroenterology and Hepatology [Internet]. 22(9):1878–1888.e10. Available from: 10.1016/j.cgh.2023.11.010 10.1016/j.cgh.2023.11.010 37993033

[CR11] Menter A, Krueger GG, Paek SY, Kivelevitch D, Adamopoulos IE, Langley RG (2021) Interleukin-17 and Interleukin-23: A Narrative Review of Mechanisms of Action in Psoriasis and Associated Comorbidities. Dermatol Ther. Adis 11:385–400. 10.1007/s13555-021-00483-2 PMC8019008 33512665

[CR12] Moher D, Liberati A, Tetzlaff J, Altman DG, Antes G, Atkins D et al (2009) Preferred reporting items for systematic reviews and meta-analyses: the PRISMA statement. PLoS Medicine 6. Available from: 10.1371/journal.pmed.1000097 10.1371/journal.pmed.1000097 PMC2707599 19621072

[CR13] Papp K, Warren RB, Green L, Reich K, Langley RG, Paul C et al (2023) Safety and efficacy of mirikizumab versus secukinumab and placebo in the treatment of moderate-to-severe plaque psoriasis (OASIS-2): a phase 3, multicentre, randomised, double-blind study. Lancet Rheumatol [Internet]. 5(9):e542–52. Available from: 10.1016/S2665-9913(23)00120-0 10.1016/S2665-9913(23)00120-0 38251498

[CR14] Raharja A, Mahil SK, Barker JN (2021) Psoriasis: a brief overview. Clin Med 21(3):170–173. Available from: 10.7861/clinmed.2021-0257 10.7861/clinmed.2021-0257 PMC8140694 34001566

[CR15] Reich K, Rich P, Maari C, Bissonnette R, Leonardi C, Menter A et al (2019) Efficacy and safety of mirikizumab (LY3074828) in the treatment of moderate-to-severe plaque psoriasis: results from a randomized phase II study. Br J Dermatol 181(1):88–95. 10.1111/bjd.17628 30734266 10.1111/bjd.17628

[CR16] Reich K, Armstrong AW, Foley P, Song M, Wasfi Y, Randazzo B et al (2017) Efficacy and safety of guselkumab, an anti-interleukin-23 monoclonal antibody, compared with adalimumab for the treatment of patients with moderate to severe psoriasis with randomized withdrawal and retreatment: Results from the phase III, double-blind, placebo- and active comparator–controlled VOYAGE 2 trial. J Am Acad Dermatol [Internet]. 76(3):418–31. Available from: 10.1016/j.jaad.2016.11.042 10.1016/j.jaad.2016.11.042 28057361

[CR17] Rendon A, Schäkel K (2019) Psoriasis pathogenesis and treatment. Int J Mol Sci 20(6):1475. 10.3390/ijms20061475 10.3390/ijms20061475 PMC6471628 30909615

[CR18] Salimi S, Yamauchi PS, Thakur R, Weinberg JM, Kircik L, Abdelmaksoud A et al (2020) Interleukin 23p19 inhibitors in chronic plaque psoriasis with focus on mirikizumab: A narrative review. Vol. 33, Dermatologic Therapy. Blackwell Publishing Inc. 10.1111/dth.13800 32530083

[CR19] Sbidian E, Chaimani A, Afach S, Doney L, Dressler C, Hua C, Mazaud C, Phan C, Hughes C, Riddle D, Naldi L, Garcia-Doval I, Le Cleach L (2020) Systemic pharmacological treatments for chronic plaque psoriasis: a network meta-analysis. Cochrane Database Syst Rev 1(1):CD011535 10.1002/14651858.CD011535.pub3 PMC6956468 31917873

