# Supplementary figures and images for: Does Mirikizumab benefit patients with plaque psoriasis? a systematic review and meta-analysis
Source: Naunyn Schmiedebergs Arch Pharmacol. 2025 Jul 10;399(1):521–30. doi: 10.1007/s00210-025-04416-0 (PMC12894435; doi:10.1007/s00210-025-04416-0)

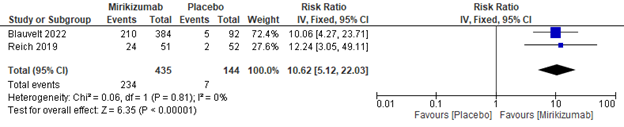

Supplement: Supplementary file 1 — (413 KB ZIP) [file 210_2025_4416_MOESM1_ESM.zip › S1.png]

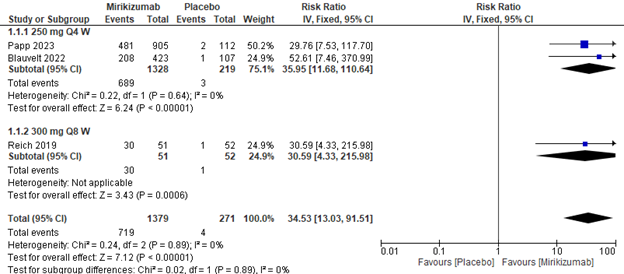

Supplement: Supplementary file 1 — (413 KB ZIP) [file 210_2025_4416_MOESM1_ESM.zip › S2.png]

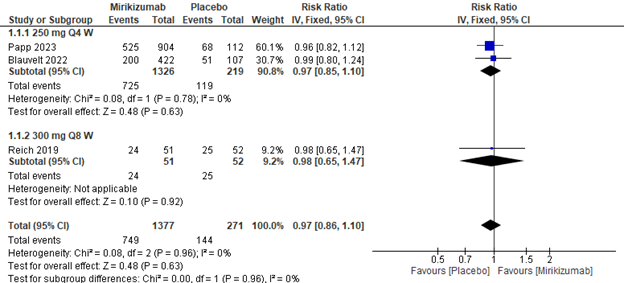

Supplement: Supplementary file 1 — (413 KB ZIP) [file 210_2025_4416_MOESM1_ESM.zip › S3.png]

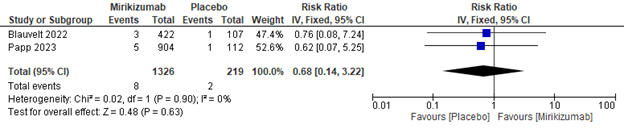

Supplement: Supplementary file 1 — (413 KB ZIP) [file 210_2025_4416_MOESM1_ESM.zip › S4.png]

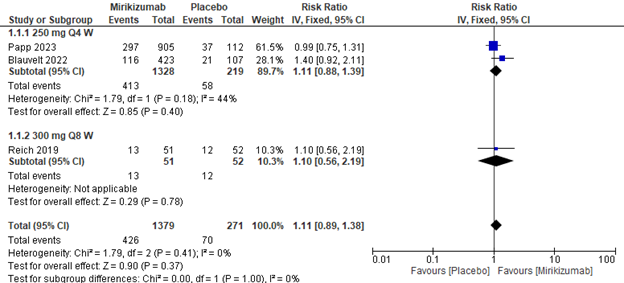

Supplement: Supplementary file 1 — (413 KB ZIP) [file 210_2025_4416_MOESM1_ESM.zip › S5.png]

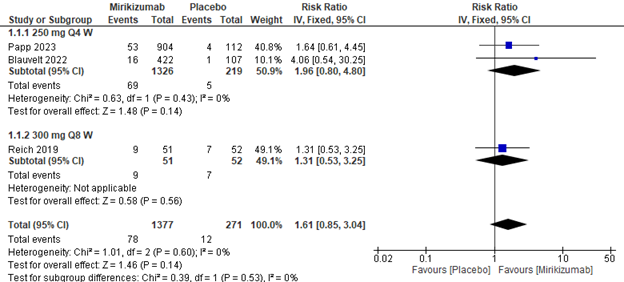

Supplement: Supplementary file 1 — (413 KB ZIP) [file 210_2025_4416_MOESM1_ESM.zip › S6.png]

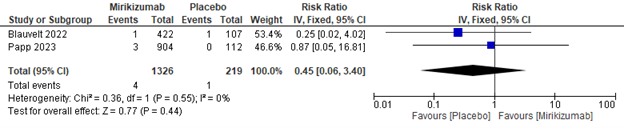

Supplement: Supplementary file 1 — (413 KB ZIP) [file 210_2025_4416_MOESM1_ESM.zip › S7.png]

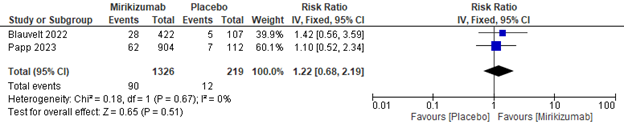

Supplement: Supplementary file 1 — (413 KB ZIP) [file 210_2025_4416_MOESM1_ESM.zip › S8.png]
